# Mechanical Properties of TWILL Carbon Fiber Fabric-Reinforced Single-Layer Thermoplastic Polyamide and Polybutylene Terephthalate-Based Composite Materials Manufactured by Hot Pressing

**DOI:** 10.3390/ma18020343

**Published:** 2025-01-14

**Authors:** Katarzyna Balcer, Dariusz Boroński

**Affiliations:** Faculty of Mechanical Engineering, Bydgoszcz University of Science and Technology, Al. Prof. S. Kaliskiego 7, 85-796 Bydgoszcz, Poland; dariusz.boronski@pbs.edu.pl

**Keywords:** composites, reinforced thermoplastic, mechanical properties, polyamide (PA6), polybutylene terephthalate (PBT), carbon fiber, strain distribution

## Abstract

This study investigates carbon fabric-reinforced thermoplastic composites produced via hot pressing, using Polyamide PA6 and Polybutylene Terephthalate (PBT) as matrix materials. These materials are increasingly utilized in the development of lightweight, high-performance, multilayer structures, such as aluminum-reinforced laminates, for automotive and aerospace applications. The mechanical properties, including tensile strength and stiffness, were systematically evaluated under varying loading conditions. The PBT-CF composite exhibited a 17% higher tensile strength and stiffness compared to the PA6-CF composite, despite the low carbon fiber content. This highlights the critical role of uniform fiber distribution in enhancing material performance. Slower loading speeds (1 mm/min) resulted in higher strength, emphasizing the influence of process parameters on mechanical behavior. Cyclic loading tests showed a gradual reduction in stiffness with increasing strain range, particularly for the CF-45° configuration. The warp and weft arrangement of the carbon fabric contributed to structural inhomogeneity but did not significantly affect the global mechanical properties. These findings demonstrate the suitability of PBT as a matrix material alongside PA6 for carbon fiber-reinforced thermoplastics, offering new possibilities for the design of advanced composite materials with tailored properties.

## 1. Introduction

Dynamic industrial development has prompted a continuous search for new, simultaneously lightweight and high-strength materials. The aviation [1,2,3], automotive [4], and maritime [5,6,7] industries are actively seeking lightweight [8,9], yet high-strength materials [10,11] due to their crucial role in reducing vehicle mass [2]. Mass reduction is a key factor influencing fuel efficiency [12], range, and vehicle performance, contributing to the reduction of harmful gas emissions into the atmosphere, in line with sustainable development requirements and emission regulations [3,4,13]. High material strength is essential to ensure the safety and reliability of structures, prioritizing these aspects in the aforementioned industries.

The revolution in the application of thermoplastic fiber-reinforced composites instead of thermosetting materials stems from several key benefits offered by thermoplastics. In contrast to thermosetting composites, thermoplastic composites possess the ability of multiple processing cycles, allowing flexibility in the production process [14]. The ability to shape and reshape components during production translates into shortened production cycle times [15], a crucial aspect in today’s dynamic industrial environment. Additionally, thermoplastics enable easy structural modification, facilitating the customization of composites for specific applications [3,6]. Thermoplastic composites are widely utilized in a variety of applications, including plates which can be utilized in structural components, automotive parts, and aerospace applications [16]; bars and rods where their products can be used in construction and sporting goods [17,18]; sheets, which can be used in the manufacturing of lightweight enclosures, panels, and other components where thermal and electrical insulation properties are also desired [19]; tubes and pipes in the oil and gas, automotive, and aerospace industries [20,21]; and cores and honeycomb structures to create core materials for sandwich structures and laminates, which are essential in aerospace and automotive applications, such as aircraft wings and body panels [21,22]. Thermoplastic composites are increasingly used in marine engineering as a substitute for steel rods, offering enhanced performance and durability in a challenging environment [23]. In addition, adhesively bonded carbon fiber-reinforced polymer systems have shown great promise for strengthening damaged steel structures [24].

For thermosetting composites, certain limitations seem to advocate for the use of thermoplastic composites [25,26]. The curing processes employed in thermosetting composites are typically more time-consuming and require precision, leading to extended production times [26]. Recycling composites containing thermosetting materials and reinforcing fibers face unique challenges associated with comprehensive component recovery [5]. Another significant drawback of thermoset composites is their brittleness, resulting from their highly cross-linked structure, which leads to low fracture toughness and limited energy absorption capacity, significantly restricting their durability and performance in load-bearing applications [27,28,29,30]. Furthermore, thermoset resins have low durability, which results in a reduced resistance to cyclic loading and environmental factors, leading to premature material failure [31,32,33]. Consequently, thermoplastic fiber-reinforced composites emerge as an attractive solution, eliminating some of these challenges and offering more flexible and efficient solutions for the mechanical engineering industry [6,18].

The production of thermoplastic fiber-reinforced composites involves the use of various polymers, each possessing unique thermal and mechanical properties. In engineering, polymers such as polyamides (PAs) [6,13,34,35,36,37], polypropylene (PP) [38], acrylonitrile-butadiene-styrene (ABS) [35], polycarbonate (PC), polyetheretherketone (PEEK), polyetherimide (PEI) [5,6,8,39], polysulfone (PES), polyethene (PE) [6], polyphenylene sulfide (PPS), and polybutylene terephthalate (PBT) [40] hold particular significance. The reinforcement of thermoplastics involves the application of various forms of reinforcements, primarily carbon, glass, or aramid fibers [8,15,26,34,41,42], influencing the final composite properties. Among the popular forms of polymer reinforcement are single fibers [43], fabrics [7,15,26,34], mats, and tapes [38,44]. Single fibers are utilized to achieve specific mechanical properties in defined directions [18]. Their precise arrangement can be controlled to optimize the composite structure for particular loads [4,35]. On the other hand, the use of fabrics allows for the flexible shaping of geometric forms, and the fabric structure facilitates even fiber distribution, affecting the excellent mechanical strength and stiffness of the composite. On the other hand, the use of fabrics allows for the flexible shaping of geometric forms, and the fabric structure facilitates even fiber distribution, affecting the excellent mechanical strength and stiffness of the composite [6,25].

Carbon fiber-reinforced thermoplastics (CFRTPs) [45,46], have been the subject of intensive scientific research due to their exceptional properties and potential engineering applications. CFRTPs combine the flexibility of thermoplastics with the exceptional strength and lightness of carbon fibers [6,45], making them an attractive alternative to traditional construction materials [3,6]. An essential feature of CFRTPs is their recyclability, including the recovery of carbon fibers [47].

Various approaches to the production of thermoplastic composites exhibit specific features tailored to different requirements and technological conditions. Examples of these methods include conventional and automated molding, as well as manual processes [6]. Among the most popular methods are injection molding [4,6,22]; vacuum bagging and oven curing [2,3,4,26,38]; compression molding and hot pressing [6,46]; lay-up, rotational molding, pultrusion, and extrusion methods [6]; and autoclave processing [2,3,4,6,26,38,48].

The research process on thermoplastic composites concerning their mechanical properties significantly benefits from molding technology as a prototyping stage before mass production. The use of molding allows for controlled and flexible small-batch production, enabling in-depth studies on various aspects of the composite structure. Molding facilitates the controlled testing of different material configurations, production processes, and forming parameters, allowing the precise adjustment of the mechanical properties of the composite to specific requirements. Additionally, it eases experimental research and analysis. Optimization at the laboratory research stage is foundational for subsequent production, eliminating potential problems and facilitating technology adaptation to a specific application. In industries where mechanical properties are crucial for the final product’s quality, initial research on thermoplastic composites through pressing is an essential step. Consequently, it enables the introduction of innovative materials to the market, harnessing the full potential of modern composite manufacturing technologies.

Despite significant research interest in carbon fiber-reinforced thermoplastics, there is a lack of literature on research results related to the production and analysis of mechanical properties of CFRTPs based on the polybutylene terephthalate (PBT) polymer. PBT is one of the most commonly used engineering polymers in the automotive and transportation industries, mainly due to its excellent dimensional stability and processing advantages [49,50]. This is the case despite slightly inferior mechanical properties, thermal stability, and wear resistance compared to other engineering polymers such as polyamide and polyoxymethylene [51]. The effective reinforcement of PBT with carbon fiber would allow for an even more attractive construction material, especially regarding its application in metal–polymer composites.

The aim of the research presented in this article was to develop an original technology and analyze the mechanical properties of a single-layer test composite, intended for the construction of thermoplastic metal laminates reinforced with carbon fibers, using an innovative composite based on the PBT polymer. The produced PBT-based composites were compared with the widely described PA6-based CFRTP composite in the literature. The analysis also examined the impact of strain inhomogeneity induced by the composite’s structure on its global mechanical properties. A key novelty of this study is the creation of a carbon fiber-reinforced composite through a pressing process, as well as an extensive analysis of strain distribution within the material. To further investigate the mechanical properties, cyclic tests with gradually increasing strain values were conducted to analyze the modulus of elasticity of the polymer materials and composites. These tests aimed to assess the durability of the materials and their ability to maintain mechanical properties under various loading conditions.

## 2. Materials and Methods

### 2.1. Raw Material

For the production of thermoplastic composites, polyamide PA6 (produced by POLITEM, Tekirdağ, Türkiye), polybutylene terephthalate PBT (produced by EPSAN, Bursa, Türkiye), and carbon fabric with a twill weave (supplied by Kordsa, İzmit, Türkiye) were utilized. Polyamide PA6 is a semi-crystalline polymer distinguished by a good combination of mechanical strength, stiffness, vibration-damping ability, and wear resistance. It also exhibits good chemical resistance but is susceptible to water absorption. Due to its favorable mechanical properties and other physicochemical characteristics, PA6 has wide applications, including the automotive and aviation industries. Literature analysis indicates numerous studies on its application in CFRTP composites produced through various methods [9,52]. Polybutylene terephthalate PBT is a semi-crystalline polymer primarily designed for the injection molding of engineering components. Key properties of PBT include high strength, elevated continuous working temperature, excellent creep resistance even at higher temperatures, high stiffness and hardness, good frictional properties, and high wear resistance. Due to its low coefficient of thermal expansion and low moisture absorption, PBT is particularly suitable for engineering applications requiring dimensional stability. The basic mechanical and physical properties of the polymers used in this study are presented in Table 1. These data were obtained from the+ manufacturers’ datasheets and pertain to materials processed by injection molding. The tests were performed at 23 °C.

Carbon fabric with a twill weave [5] allows the formation of complex geometric shapes, facilitating the thermoforming of preproduced CFRTP composite sheets, subsequently used for thermoplastic fiber metal laminate (TFML). In the study, a fabric made from carbon fibers intended for thermoplastic composites, including products for aerospace and automotive applications, was employed. The basic mechanical properties of carbon fibers and carbon fabric are presented in Table 2 and Table 3.

### 2.2. Manufacturing

Before the composite manufacturing process, both polymers and carbon fabric underwent drying according to Table 4. The drying conditions for the polymers were determined based on widely available information regarding the selected materials, while the drying conditions for the carbon fabric were established through numerous preliminary studies to ensure optimal preparation for composite production.

CFRTP composite test plates were produced using the hot pressing technique of two polymer layers with carbon fabric placed between them.

**Figure 1:**
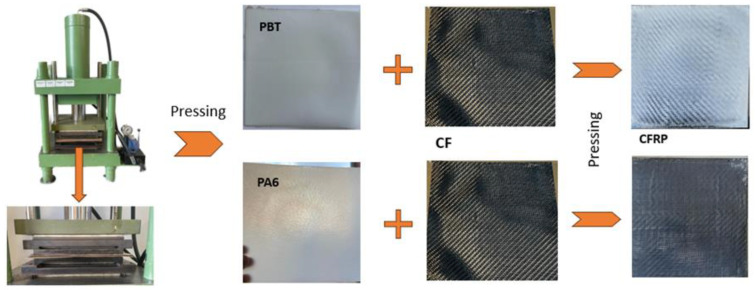
CFRTP panel manufacturing process.

Firstly, PA6 and PBT layers with a thickness of 0.5 mm were produced. For this purpose, on a 1 mm thick stainless-steel plate with an anti-adhesive insulating layer placed in a press with heated platens, a steel frame with a height of 0.5 mm was mounted (Figure 1). A measured amount of polymer granules, 22.4 g PA6 and 26.2 g PBT, allowing the formation of a 0.5 mm thick plate, was introduced into the frame. By applying press pressure under the specified temperature conditions of the platens, the granules became plasticized and formed into a plate with dimensions defined by the metal frame. After the process, the plates were left inside the metal frame.

Subsequently, a layer of carbon fiber fabric was introduced between two frames with polymer plates and subjected to the pressing process under specified temperature and pressure conditions. The process parameters for the production of composite plates are presented in Table 5. The processing temperature was determined based on widely available information regarding the selected polymers, while the pressure and time were optimized through numerous preliminary experiments, aimed at identifying the most suitable conditions for the composite production process.

### 2.3. Sample Preparation

**Figure 2:**
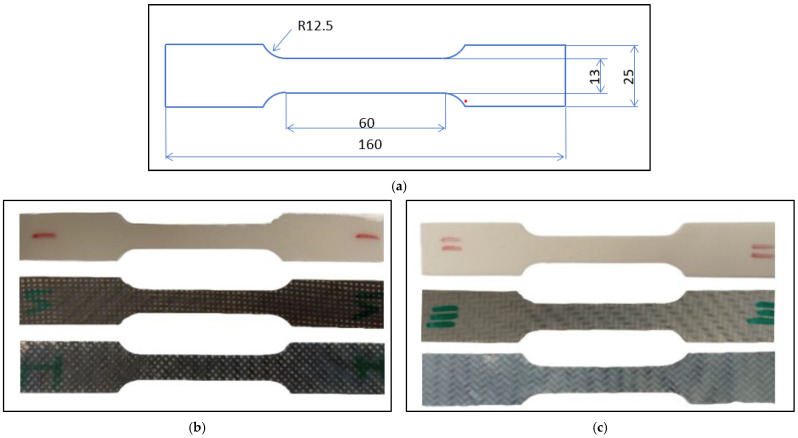
Samples for static properties testing: (**a**) sample dimensions, (**b**) PA6-CF samples, (**c**) PBT-CF samples.

Polymer plates and the composites produced in the aforementioned way were used to create samples for mechanical property testing. Photos of the samples and a drawing with their dimensions are shown in Figure 2. The sample geometry [53] adopted for subsequent research on thermoplastic fiber metal laminate (TFML) composites, based on the developed PA6-CF and PBT-CF composites, was employed. Samples were cut from the plates using milling.

The produced samples were evaluated for carbon fiber filling in their cross-sectional area to ensure uniform fiber participation in load distribution.

### 2.4. Test Methods

Static mechanical property tests were conducted according to the EN ISO 527-1:2019 [54] at displacement speeds of 1 mm/min and 10 mm/min. The 10 mm/min speed was selected as typical for polymers, while 1 mm/min allows for future comparisons with tests on sandwich structures and TFMLs, in which these materials will serve as constituent components.

Additionally, to analyze the modulus of elasticity of polymer materials and composites, cyclic tests with gradually increasing strain values were conducted with the use of servohydraulic INSTRON 8502 testing machine (INSTRON, Norwood, MA, USA). Samples with a constant width of 12.8 mm and length of 200 mm were used for the tests.

The tests were conducted in the displacement control mode at a rate of 2 mm/min. The modulus was determined from stress–strain curves within a linear range from 0.05% to 0.20% strain. In the cyclic loading–unloading tests (L–UL), the samples underwent steps of increasing strain, starting from 0.25% until failure with the step of 0.1%. After each loading step, the samples were unloaded to 100 N, and a low-level (up to 0.25% strain range) L–UL step was performed from which the modulus was determined. The investigation of tensile modulus degradation aims to assess the durability of materials and their capacity to sustain mechanical properties under various loading conditions. Samples with a constant width of 12.8 mm and length of 200 mm were used for the tests.

**Figure 3:**
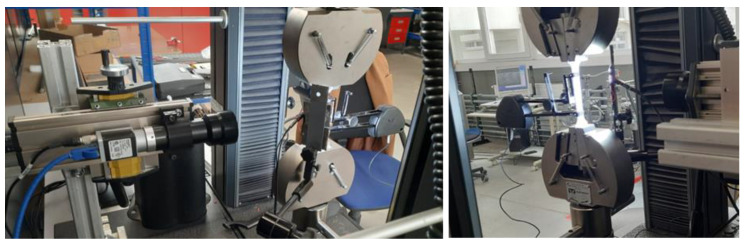
The Digital Image Correlation (DIC) measurement setup.

Strain in the samples was determined based on transverse displacement measurements (a standard test implemented in the Zwick Roell machine control system) and using an external extensometer attached to the sample (INSTRON, Norwood, MA, USA). Additionally, for the analysis of deformations and their distribution in the samples during strength tests, the method of digital image correlation utilized in the research was outlined in [55]. The strain measurement was performed on the basis of an image of the specimen’s natural surface without applying additional markers. High-quality telecentric VS Technology lenses with micrometric optical resolution and high-resolution Basler Ace cameras were used to observe the specimen image (Figure 3).

The density analysis of the developed composites was performed utilizing a helium pycnometer to ensure a high accuracy and reliability of the measurements. The specific instrument employed for this purpose was the Pycnomatic Thermo Scientific (Milan, Italy), a device well-regarded for its precision in determining material densities. The density analysis was conducted to compare the obtained results with the values provided in the material’s datasheet and to subsequently utilize these data as key parameters in the design and analysis of laminates.

## 3. Results

### 3.1. Static Mechanical Properties

The static mechanical properties of the base materials (PA6 and PBT) and developed composites (PA6-CF and PBT-CF) were examined using two independent testing systems with different loading speeds: 10 mm/min and 1 mm/min. Static mechanical properties of PA6-CF and PBT-CF composites were investigated on samples taken at two fabric orientation angles relative to the longitudinal axis of the specimen: 0° (PA6-CF-0/90° and PBT-CF-0/90°) and 45° (PA6-CF-45° and PBT-CF-45°).

**Figure 4:**
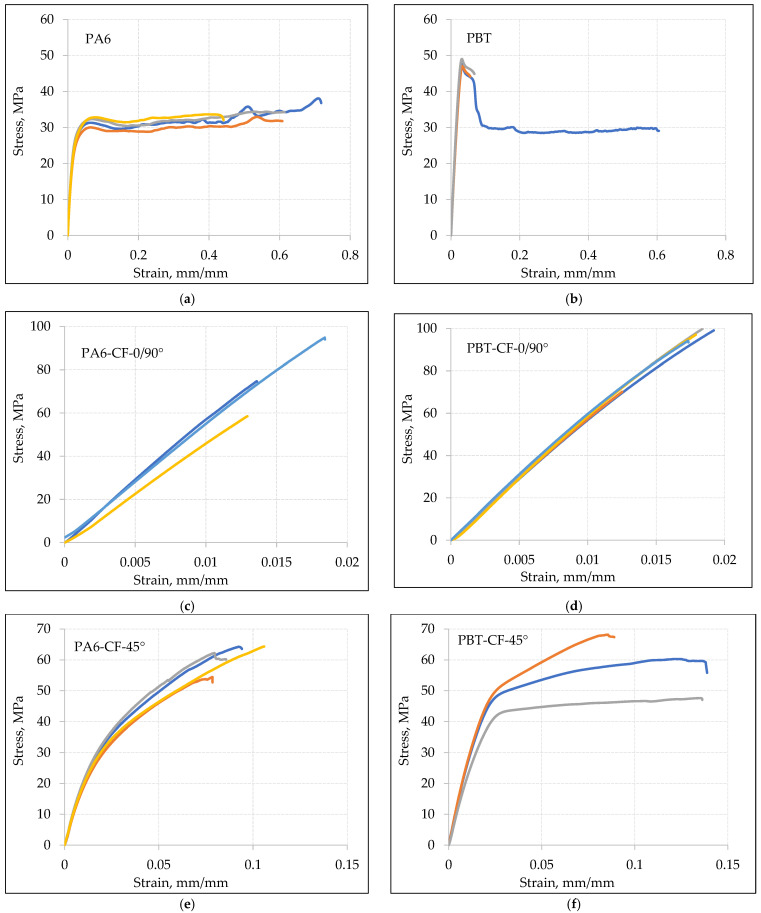
Static tensile curves for loading speed v = 10 mm/min: (**a**) PA6, (**b**) PBT, (**c**) PA6-CF-0/90°, (**d**) PBT-CF-0/90°, (**e**) PA6-CF-45°, (**f**) PBT-CF-45°. Each color represents a different sample.

**Figure 5:**
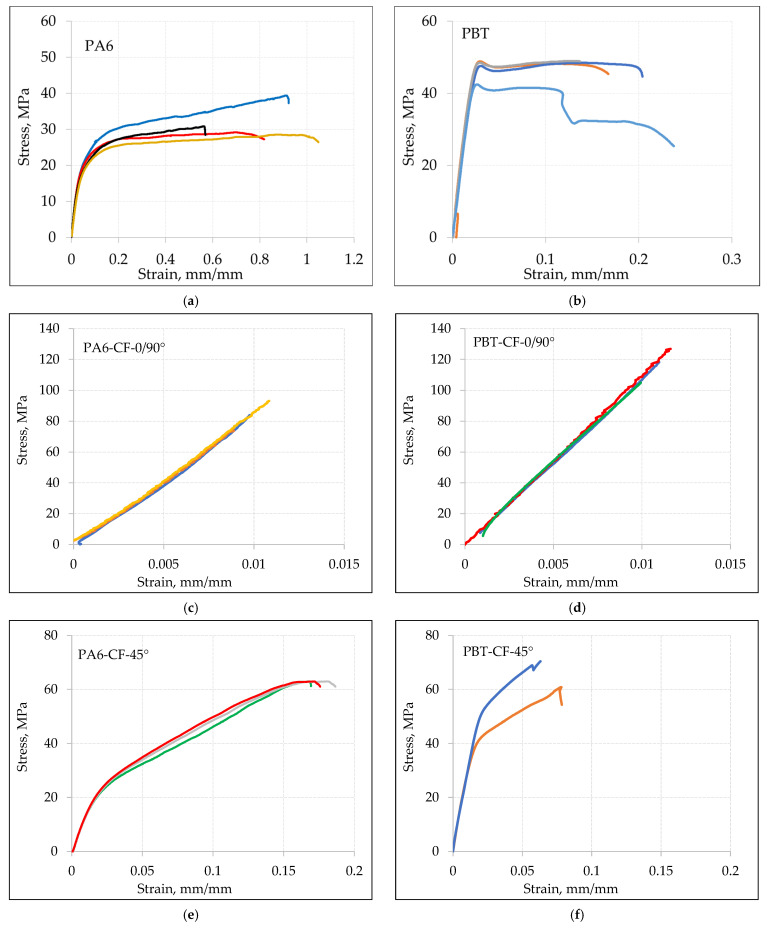
Static tensile curves for loading speed v = 1 mm/min: (**a**) PA6, (**b**) PBT, (**c**) PA6-CF-0/90°, (**d**) PBT-CF-0/90°, (**e**) PA6-CF-45°, (**f**) PBT-CF-45°. Each color represents a different sample.

Sample stress–strain curves for various types of samples obtained at a displacement speed of v = 10 mm/min are shown in Figure 4, and for a displacement speed of v = 1 mm/min in Figure 5.

Based on the analysis of tensile curves, the mean values of mechanical properties for base materials and composites PA6-CF and PBT-CF are compiled in Table 6 (at a loading speed of v = 10 mm/min) and in Table 7 (at a loading speed of v = 1 mm/min).

### 3.2. Tensile Modulus

**Figure 6:**
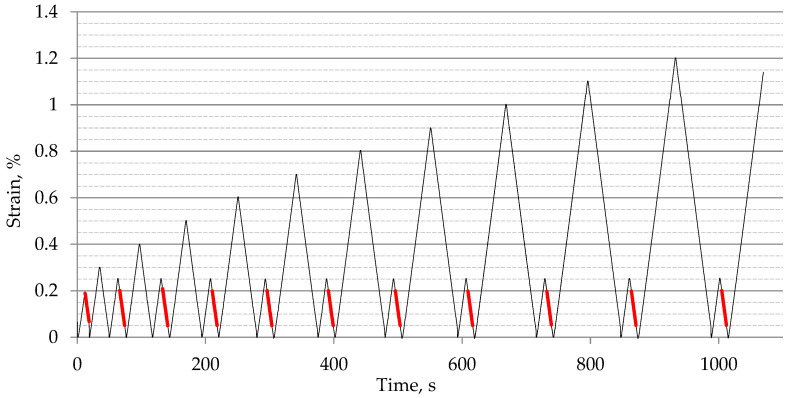
Schematic representation of the loading applied during the determination of the tensile modulus. Strain range considered during the determination of the modulus value has been marked in red.

In addition to the studies on mechanical properties, an analysis of the longitudinal elastic modulus was conducted under conditions of cyclically varying load, with an increasing strain range. Figure 6 shows a representative curve of the changes in strain values in the PBT-CF-45° sample, with the strain range considered during the determination of the modulus value.

**Figure 7:**
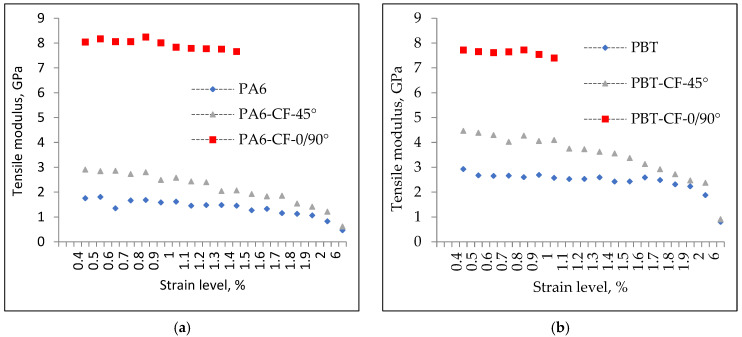
Values of tensile modulus for PA6, PA6-CF-45°, and PA6-CF-0/90° samples (**a**) and PA6, PBT-CF-45°, and PBT-CF-0/90° samples (**b**) determined in a test with increasing strain values.

The tensile modulus values determined in the above manner for PA6, PA6-CF-45°, PA6-CF-90°, PBT, PBT-CF-45°, and PBT-CF-90° for the first loading cycle are summarized in Table 8, and the course of their changes after implementing subsequent levels of deformation is presented in Figure 7.

### 3.3. Density

The densities of the base materials and the investigated composites, determined using a helium pycnometer, are presented in Table 9. It was observed that the density values do not differ significantly, whether the samples were produced using the injection molding method (as provided by the manufacturer) or created through the pressing process. These results suggest that the pressing process does not have a significant impact on the composite density compared to injection molding-based production methods.

### 3.4. Displacement Distribution

**Figure 8:**
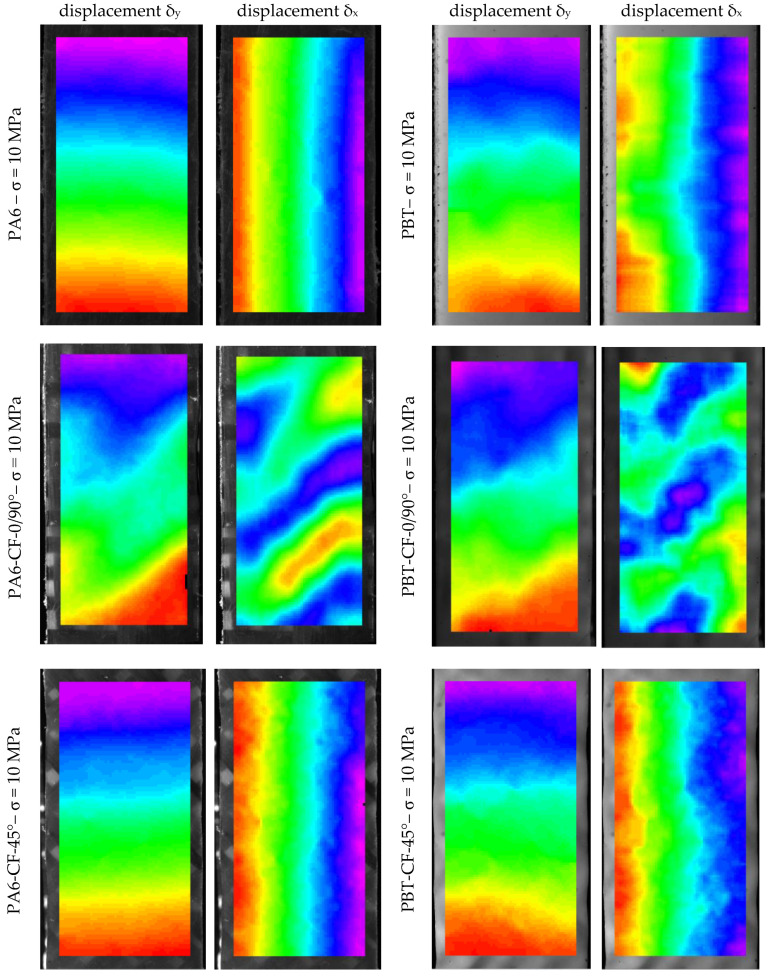
Examples of displacement distributions in specimens for the direction in line with the load direction (δ_y_) and for the direction transverse to the load direction (δ_x_). The colors represent the displacement differences in the observed field of the specimen under the applied loads.

In order to assess the influence of the heterogeneity of the structure of the developed CFRTPs on their mechanical properties, strain distributions in the specimens were analyzed using digital image correlation. The deformations were determined from the displacements, the sample distributions for which were measured for each specimen type for an equal tensile test stress value and are shown in Figure 8.

## 4. Discussion

### 4.1. Comparative Analysis of Mechanical Properties of Composites

**Figure 9:**
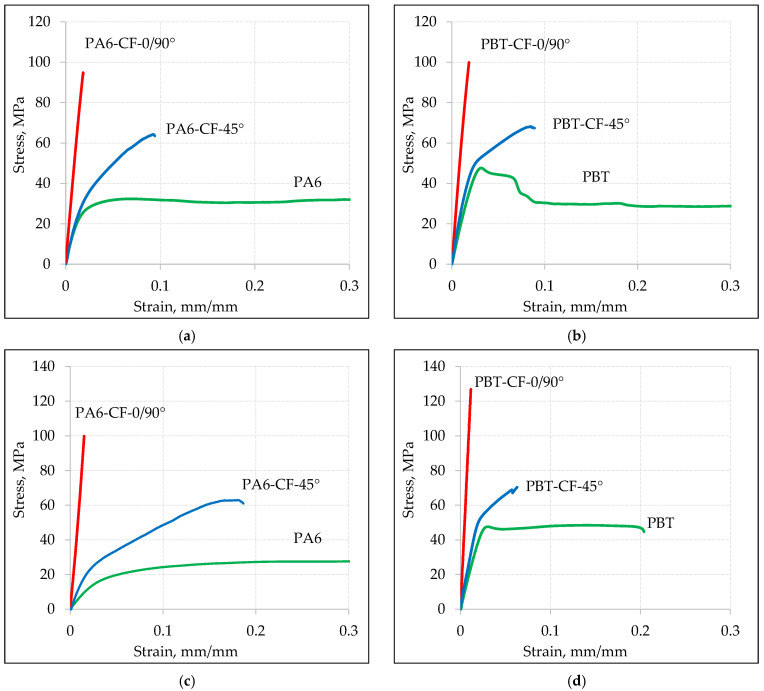
Comparison of static tensile stress–strain curves for base materials and composite materials at loading speeds of 10 mm/min (**a**) PA6, (**b**) PBT, and at a loading speed of 1 mm/min (**c**) PA6, (**d**) PBT.

The comparison of stress–strain curves for PA6 and PBT polymers, with and without carbon fiber reinforcement, is shown in Figure 9 for a loading rate v = 10 mm/min (Figure 9a,b) and v = 1 mm/min (Figure 9c,d). As expected, in all cases, the strength of the carbon fiber-reinforced samples noticeably increased, with a simultaneous decrease in elongation.

In the case of samples with a 0/90° fiber arrangement, the stress–strain relationship was dominated by the mechanical properties of carbon fibers. The maximum elongation in this case is close to the strain at failure for the carbon fiber, which is 1.5%. Samples with a 45° fiber arrangement exhibit higher relative elongation but, at the same time, lower strength, which is a result of a different load transfer mechanism. While in samples with a 0/90° fiber arrangement, the load is transferred in a parallel system through the carbon fabric and the polymeric matrix, in samples with a 45° fiber arrangement, a mixed parallel serial loading character of the fibers occurs. This, on one hand, increases the material’s ability to deform, but on the other hand, strengthens the composite to a lesser extent.

Quantitative comparison of the impact of reinforcing PA6 and PBT with carbon fabric is presented in Table 10. It shows an increase in the strength of PA6-CF-0/90° samples by approximately 136% to 178% (depending on the loading speed) compared to PA6.

A similar effect was achieved with PBT, where the strength increased by approximately 92% for v = 10 mm/min to about 145% for v = 1 mm/min.

Significantly lower increases in strength were noted in the case of the 45° fiber arrangement. In the case of PA6, regardless of the loading speed, the strength increased by about 76–78. For PBT, the strength increased by about 40% for v = 10 mm/min and about 42% for v = 1 mm/min.

Considering the research results, it is important to note that the obtained increases in strength are a result of only a few percent of carbon fibers in the cross-sectional area of the sample, which is discussed in more detail in the later part of the chapter.

The influence of carbon fiber fabric reinforcement on the tensile modulus of PA6 and PBT exhibited a similar character, with its quantitative description presented in Table 11. A significantly greater impact on the change in stiffness occurred for the 0/90° configuration, amounting to approximately 360% for PA6 and about 165% for PBT, while for the 45° arrangement, the relative increase in stiffness was 65% for PA6 and 55% for PBT.

**Figure 10:**
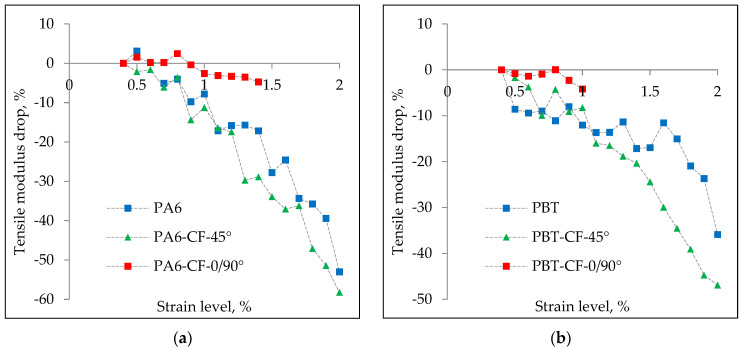
Variations in tensile modulus values obtained from a progressively increasing load test for composites made from PA6 (**a**) and PBT (**b**).

The cyclic loading applied in the tensile modulus studies also allowed for the determination of the degree of stiffness degradation of the composites and polymer materials. The analysis was conducted for a strain range up to 2%, with results shown in Figure 10. The trend of tensile modulus changes exhibits a similar character for both polymers, although it is somewhat more pronounced for composites based on PA6. The modulus value decreases progressively with the implementation of successive strain levels, with the largest drop occurring at 2% strain. For carbon fiber-reinforced polymers in the 45° configuration, the reduction reached approximately 50% for PBT and 60% for PA6.

**Figure 11:**
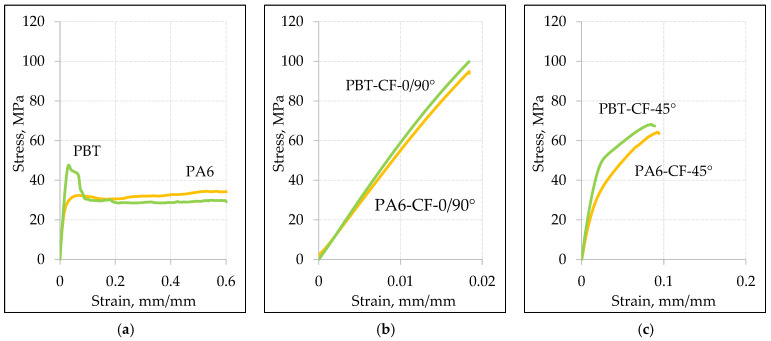

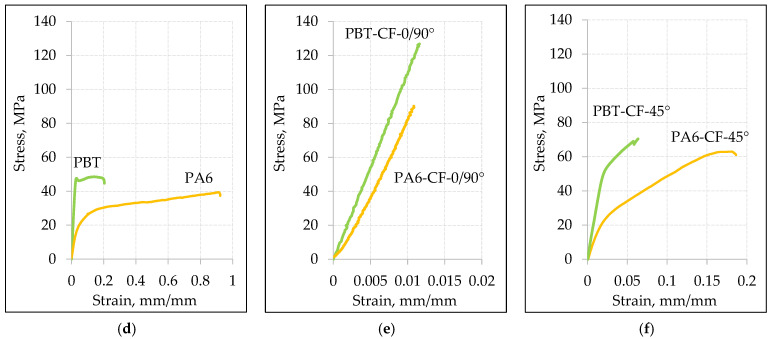
Comparison of tensile stress–strain curves for base materials PA6 and PBT (**a**), composites in the 45° direction (**b**), and 0/90° direction (**c**) at a loading speed of 10 mm/min, as well as tensile stress–strain curves for base materials PA6 and PBT (**d**), composites in the 45° direction (**e**), and 0/90° direction (**f**) at a loading speed of 1 mm/min.

Considering the lack of literature on the use of PBT in CFRTP composites [11,56,57,58,59,60,61,62,63,64,65,66,67], it was significant to compare the mechanical properties of the developed PBT-CF composite with the better-known and described PA6-CF composite. For this purpose, tensile stress–strain curves were plotted for unreinforced material and samples were taken for both the 0/90° and 45° fiber arrangements (Figure 11). In each of the analyzed cases, the mechanical properties of PBT-CF achieved higher values than PA6-CF. This applies to both loading speeds, but both the tensile strength and stiffness of the PBT-CF composite samples noticeably achieve higher values at a speed of 1 mm/min.

The results of a quantitative comparison of the differences in the mechanical properties of PBT-CF and PA6-CF composites are presented in Table 12. The tensile strength of PBT-CF for v = 1 mm/min is higher by approximately 25% compared to the tensile strength of the PA6-CF composite for the 0/90° arrangement and by about 15% for the 45° arrangement. In the case of a speed of v = 10 mm/min, the difference is less pronounced, amounting to about 12% for the 0/90° arrangement and 8% for the 45° arrangement.

Also, in the case of the modulus of elasticity, the comparison results favor PBT-CF, where the stiffness increase reaches values ranging from 55 to 65%. Only in the case of the CF 0/90° configuration does the initial stiffness of the composites exhibit a similar value.

Taking into consideration the intended use of the investigated composites, the analysis also involved the value of the strength/weight ratio (SWR) described by the relationship and the elasticity/weight ratio (EWR):

(1) $$SWR=\frac{\sigma_{m}}{\rho }$$

(2) $$EWR=\frac{E_{t}}{\rho }$$

The calculated values have been included in Table 13.

Comparison of the data from Table 12 allows us to observe similar values of SWR for both composites. Substantial differences arise in the case of the EWR indicator, where the composite with PBT achieves about a 20% increase compared to the PA6 composite.

### 4.2. Strain Distribution

In the test samples, based on the displacement distributions measured by digital image correlation (during the tensile test), the strain distributions in the direction of the loading axis ε_y_ and in the direction transverse to the loading axis ε_x_, were determined according to the relationships:

(3) $$\varepsilon_{y}=\frac{\partial \delta_{y}}{\partial y},\;\varepsilon_{x}=\frac{\partial \delta_{x}}{\partial x}$$

**Figure 12:**
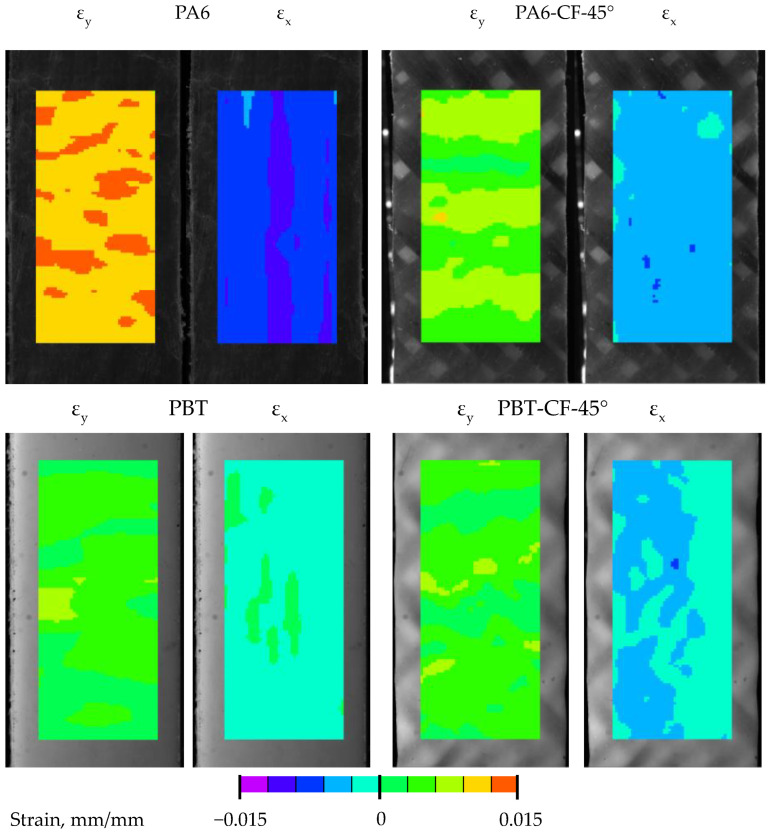
Strain distributions in PA6, PA6-CF-45°, PBT, and PBT-CF-45° samples.

In the samples PA6 and PBT (base materials) and PA6-CF45° and PBT-CF45° (fabric-reinforced polymers in a 45° fiber arrangement), the strain distributions are generally uniform, as shown in the examples in Figure 12. For easier comparison of the determined strain distributions, the same color scale was used for all samples. As expected, the strains ε_y_ have positive values over the entire measured area and the strains ε_x_ are compressive strains. However, for specimens with a 0/90° fiber arrangement in a polymer matrix, there is a significant change in the strain distribution caused by their axial loading.

**Figure 13:**
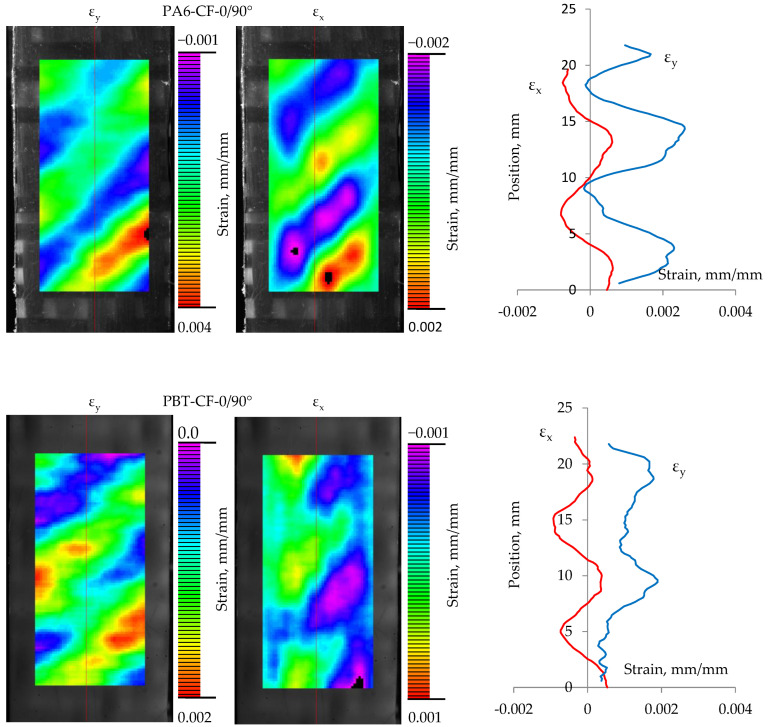
Strain distributions in PA6-CF-0/90° and PBT-CF-0/90° samples. The red line shows ε_x_ strains while blue line shows ε_y_ strains.

As shown in Figure 13, the strain distributions in specimens with a 0/90° fiber system are more complex in nature. For both ε_y_ and ε_x_ strains, their values vary along the length of the specimen in a quasi-sinusoidal manner. As expected, they have positive values in the case of ε_y_ deformations. Less obvious is the nature of the transverse deformations ε_x_. For both PA6-CF and PBT-CF specimens, their values indicate the simultaneous presence of compression and tension areas in the specimens. From the comparison of the strain maps with the structure of the fiber bundles in the fabric, it is evident that the strain concentration is related to the arrangement of warps and wefts. Furthermore, strains of the same nature (tensile or compressive) are arranged obliquely with a specific spacing related to the arrangement of the bundles.

This character of deformation is related to the specificity of the TWILL 2 × 2 weave of the carbon fiber fabric used. The deformation distributions obtained are consistent in their form with the results of experimental and numerical studies of the epoxy-carbon composite described in the paper [68]. According to its authors, the highest deformation occurs at the intersections of weft and warp yarns. The reason the localized deformation occurs in the resin-rich region is because the modulus there is much lower than in the fiber zone. In the case of transverse deformation, its concentrations are localized in the matrix areas. According to the authors, this is because warp yarns play the dominant role in load transfer, while the contribution of weft yarns is negligible. For this reason, the transverse deformations in the warp yarns are much smaller than those in the weft yarns.

While the cyclic character of the deformations in the specimens can be explained in this way, it does not fully answer the question regarding the occurrence of significant tensile deformations in the transverse direction, which are equal in magnitude to compressive deformations.

Analysis of the strain maps in the CF-0/90° composite specimens reveals another peculiar feature of the composite. The areas of the specimens with the highest values of positive transverse strain (ε_x_) overlap with the highest longitudinal strain (ε_y_). Such properties are characteristic of the group of materials referred to as metamaterials, but in the case of the composites analyzed they are only local and do not translate into their global properties, including, above all, the Poisson’s number.

### 4.3. Poisson’s Ratio

Application of flat samples and the method of digital image correlation enabled the determination of Poisson’s ratio ν for all the analyzed cases on the base of the determined strains ε_x_ (in the direction transverse to the direction of loading) and ε_y_ (along the direction of loading) during loading.

**Figure 14:**
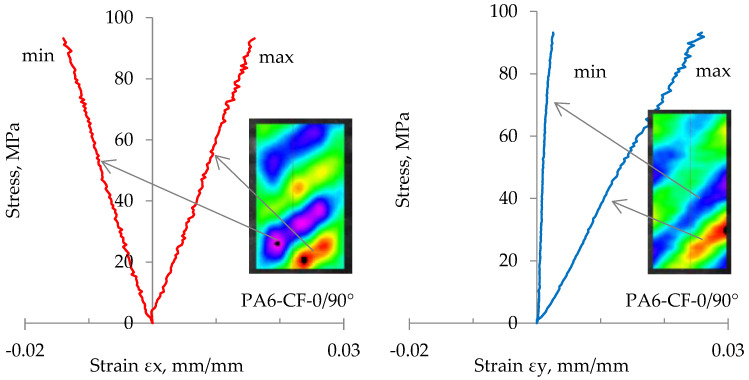
Course of variation of maximum and minimum strain values ε_x_ and ε_y_ in sample PA6-CF-0/90°. The red line is strain ε_x_ and blue line is strains ε_y_.

As noted in the previous chapter, inhomogeneities in strain distributions can affect local Poisson’s number values. This is shown in Figure 14, using the example of strain results in the zones with the smallest and largest strains in the PA6-CF-0/90° sample.

**Figure 15:**
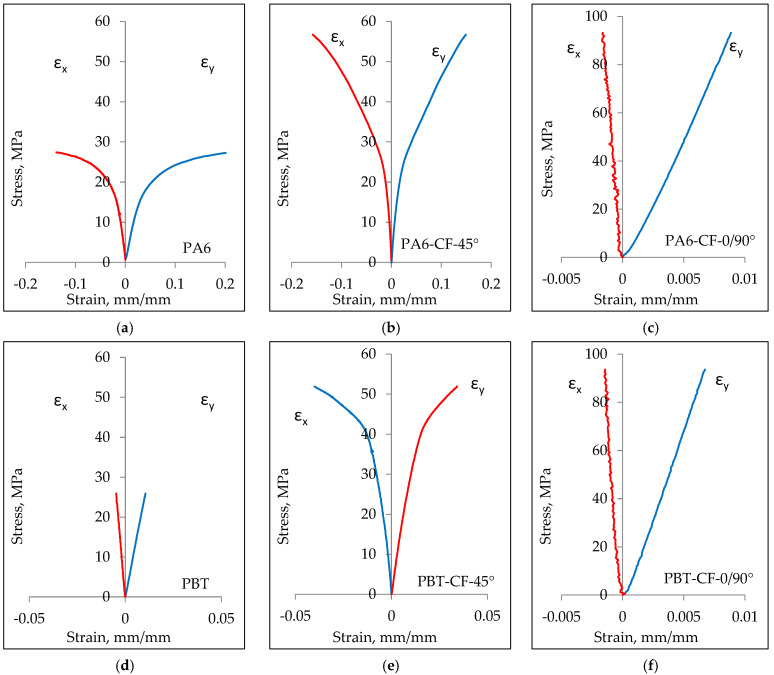
Changes in average strain values εx and εy in samples PA6 (**a**), PA6-CF-45° (**b**), PA6-CF-0/90° (**c**), PBT (**d**), PBT-CF-45° (**e**), PBT-CF-0/90° (**f**).

**Figure 16:**
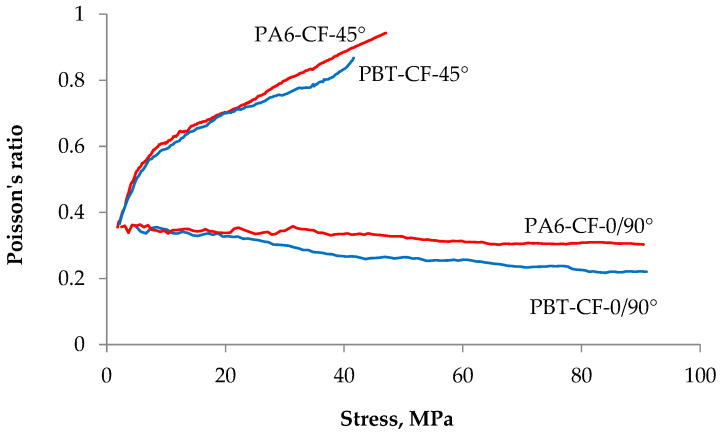
Dependence of Poisson’s ratio on tensile stress value.

For this reason, in order to assess the influence of strain inhomogeneity on the global Poisson’s number values, the Poisson’s number values were determined by averaging the strains from the entire surface of the specimens subjected to DIC measurement (Figure 15). The Poisson’s number values determined from these vary with the level of loading, as illustrated in Figure 16, using specimens CF-45° and CF-0/90°.

For low loads, the Poison’s ratio values of the PA6-CF and PBT-CF samples do not differ from their values for the matrix material, which are around 0.4 (0.38–0.45 for PA6 and 0.39–0.41 for PBT). However, as the load increases, the Poisson’s ratio changes, but in a different way for samples CF-45° and CF-0/90°. Arranging the carbon fabric at an angle of 45° with respect to the load direction results in a strong and rapid increase in Poisson’s ratio, while the Poisson’s ratio value decreases uniformly when the fabric is arranged longitudinally. The decrease in the Poisson’s ratio value may be due to the warp yarns gradually approaching each other, and thus, reducing the influence of the warp on the transverse strain value.

At the same time, it can be noted that the type of matrix material did not significantly affect the Poisson’s ratio values of the composites tested.

The observed values of Poisson’s ratio in the studies, which significantly exceed the range accepted for homogeneous, isotropic, and elastic materials (from 0 to 0.5), are corroborated by other research, including those described in the publication [69].

According to the authors, this is particularly significant for heterogeneous materials due to the specificity of their structure, which is often the case with woven fabrics. Consequently, the transverse strain during stretching is less than the longitudinal strain, which is not observed in the case of a less dense 2/2 bias sample. In the described studies, the values of Poisson’s ratio ranged from 0.07 to 0.78 in the warp direction and from 0.46 to 1.39 in the weft direction, depending on the samples considered. For strains outside the principal axes, the obtained Poisson’s ratio reached values as high as 3.9. The authors attribute this to the specific structure of the woven fabric. When the woven fabric is stretched in a direction outside the principal axis, the mutually perpendicular yarn systems rotate relative to one another, leading to the closure of the porous structure. This results in a reduction in the material’s volume under tensile stress, causing very high transverse strain relative to the tensile strain, and consequently, high values of Poisson’s ratio.

## 5. Conclusions

The project objectives have been successfully achieved. Thermoplastic-based composites—the well-known polyamide PA6 and the innovative polymer PBT—have shown promising applications as components in both sandwich structures and thermoplastic metal–fiber laminates. The analysis of the results led to the following conclusions:

- Despite the low percentage of carbon fibers, a significant increase in strength was observed, suggesting that precise fiber distribution is a crucial factor influencing the final mechanical properties of the composite.
- Higher tensile strength and stiffness values were observed in the PBT-CF composite compared to PA6-CF. This is noteworthy, especially considering the lack of literature on PBT applications in CFRTP composites, suggesting potential innovative uses of this material in advanced composite structures.
- Higher sample strength was observed at a lower loading speed (v = 1 mm/min). It can be speculated that rheological processes and interactions between carbon fibers and the thermoplastic matrix undergo more controlled shaping at a slower deformation rate.
- Cyclic loading of the tested composites leads to a gradual decrease in their stiffness with an increase in the strain range, which is most pronounced in the case of the CF-45° configuration. The inhomogeneity of strain distributions associated with the warp yarn and weft yarn arrangement in the carbon fabric used did not result in significant changes in the global mechanical properties of the composites tested.

In future studies, the same manufacturing technology will be employed to produce composites with reduced thickness, utilized as components in thermoplastic metal–fiber laminates.

## Figures and Tables

**Table 1:** Physical and mechanical properties of polymers used in the study.

Polymer	Density, g/cm^3^	Tensile Strength at Break, MPa	Elongation at Break, %	Young Modulus, MPa
PA6	1.12–1.15	65–85	10–100	2500–3500
PBT	1.31	60	200	2900

**Table 2:** Properties of carbon fibers.

Carbon Fiber Type	Density	Filament Diameter	Filament Count	Tensile Strength	Tensile Modulus	Strain at Failure
T300	1.76 g/cm^3^	0.007 mm	3000	3530 MPa	230 GPa	1.5%

**Table 3:** Properties of carbon fabric.

Type	Area Weight	Thickness	Set of Warp	Set of Weft
TWILL 2/2	160 g/m^2^	0.34 mm	400/m	400/m

**Table 4:** The drying parameters of polymer granulate and carbon fabric.

Material	Drying Temperature, °C	Drying Time, h
Polyamide PA6	80–110	4–5
Polybutylene terephthalate PBT	110–130	2–4
Carbon fabric	80	1

**Table 5:** Technological parameters of pressing.

Material	Temp., °C	Pressure, MPa		Process Time, s
PA6	220	Both polymers	no pressure	120
			1–2	60
PBT	225		5	
			20	
PA6-CF	220	Both CFRTPs	no pressure	120
PBT-CF	225		5–6	
			20	

**Table 6:** Mechanical properties determined at a loading speed of v = 10 mm/min.

Material	σ_m_, MPa	Ɛ_b_, %
PA6	34.7 ± 2.4	70 ± 17
PA6-CF-0/90°	81.85 ± 19	1.7 ± 0.4
PA6-CF-45°	61.9 ± 3.9	8.85 ± 1.3
PBT	47.9 ± 1	5.72 ± 0.5
PBT-CF-0/90°	92.1 ± 11	1.8 ± 0.3
PBT-CF-45°	67.0 ± 14	13.8 ± 3

**Table 7:** Mechanical properties determined at a loading speed of v = 1 mm/min.

Material	σ_m_, MPa	Ɛ_b_, %
PA6	33.1 ± 4	83.2 ± 20
PA6-CF-0/90°	92.1 ± 7	1.1 ± 0.5
PA6-CF-45°	58.3 ± 3.6	18.2 ± 2.5
PBT	46.9 ± 3.7	14.7 ± 3.5
PBT-CF-0/90°	115.2 ± 11.6	1.1 ± 0.1
PBT-CF-45°	66.8 ± 7.5	7.8 ± 1.6

**Table 8:** Tensile modulus.

	PA6	PA6-CF-45°	PA6-CF-0/90°	PBT	PBT-CF-45°	PBT-CF-0/90°
Tensile modulus, GPa	1.8 ± 0.03	2.9 ± 0.18	8 ± 0.32	2.9 ± 0.05	4.5 ± 0.26	7.7 ± 0.15

**Table 9:** Densities of base materials and composites.

Material	PA6	PBT	PA6-CF	PBT-CF
Density, g/cm^3^	1.13 ± 0.01	1.31 ± 0.02	1.18 ± 0.03	1.34 ± 0.03

**Table 10:** Comparison of the ultimate tensile stress of base materials and composites.

	[(σ_m_PM-CF-0/90°_ − σ_m_PM_)/σ_m_PM_]·100%	
	10 mm/min	1 mm/min
PM: PA6	135.9%	178.2%
PM: PBT	92.3%	145.6%
	[(σ_m_PM-CF-45°_ − σ_m_PM_)/σ_m_PA6_]·100%	
	10 mm/min	1 mm/min
PM: PA6	78.4%	76.1%
PM: PBT	39.9%	42.4%

**Table 11:** Comparison of the tensile modulus of base materials and composites.

	[(E__PM-CF-0/90°_ − E__PM_)/E__PM_]·100%
PM: PA6	357.1%
PM: PBT	165.5%
	[(E__PM-CF-45°_ − E__PM_)/E__PM_]·100%
PM: PA6	65.7%
PM: PBT	55.2%

**Table 12:** Comparison of mechanical properties of PBT-CF and PA6-CF.

	[(σ_m_PBT_ − σ_m_PA6_)/σ_m_PA6_]·100%		[(E__PBT_ − E__PA6_)/E__PA6_]·100%
Loading Rate	v = 10 mm/min	v = 1 mm/min	
Without CF	38.0%	41.7%	65.7%
CF 0/90°	12.5%	25.1%	−3.8%
CF 45°	8.2%	14.6%	55.2%

**Table 13:** SWR and EWR index values.

Material	PA6-CF-0/90°	PA6-CF-45°	PBT-CF-0/90°	PBT-CF-45°
SWR, MPa·cm^3^/g	65.4	52. 5	68.7	43.8
EWR, MPa·cm^3^/g	2331	1178	3082	1425

## Data Availability

The original contributions presented in the study are included in the article, further inquiries can be directed to the corresponding author.
